# Plasticity properties and undrained strength of bio-enzyme treated black-cotton soils

**DOI:** 10.1038/s41598-025-21043-0

**Published:** 2025-10-23

**Authors:** T. Rajani Priya, H. B. Nagaraj, S. Muguda

**Affiliations:** 1 Department of Civil Engineering, B.M.S College of Engineering, Bangalore, 560 019 Karnataka India; 2 Department of Engineering, Durham University, Durham, DH13LE UK

**Keywords:** Black cotton soil, Enzymes, Atterberg limits, Undrained strength, Civil engineering, Environmental impact

## Abstract

For Black cotton (B.C.) soils many alternate stabilization techniques are being suggested by researchers from time to time, which partially address the problem of volumetric instability with moisture changes. Of late, with the success of the enzymatic stabilization of kaolinitic soils, researchers have tried to extend the application of enzymatic stabilization to B.C. soils. The reported information in the literature on index properties of enzyme treated B.C. soils is very intriguing. In this study, two different formulations of a commercially available TerraZyme (TZ 5X and TZ 11X) with four selected dosages were used to understand the changes in index as well as undrained strength properties with ageing of two B.C. soils selected from different geological locations. Contrasting changes in plasticity properties on enzymatic treatment using TZ 5X with ageing are observed for the two B.C. soils used in the present study. However, TZ 11X has shown to have a slight reduction in liquid limit and an increase in shrinkage limit. As index properties are inferential properties of soils, changes in plasticity properties by using enzymes may not be the only indication of the effectiveness of enzymatic stabilization. Enzyme treated soils yielded a marked improvement in undrained strength indicating a better parameter to assess the effectiveness of enzymatic stabilization of B.C Soils.

## Introduction

One of the main challenges in road construction and its performance is the diversity of soil types along highway segments, leading to varied physical and engineering properties. So, for a reliable and improved performance and long-term stability of the subgrade soils, they need to be adequately stabilized if their properties are found to be unsatisfactory. Cement and lime have been conventionally utilized as stabilizers to enhance the engineering properties of subgrade soils^1,2^. Of late, researchers and practicing engineers have realized the impact of using conventional stabilizers on the environment, which are highly energy-intensive and lead to the production of Greenhouse Gases (GHGs)^3–5^. As a substitute, the use of environmentally friendly stabilizers derived from organic matter in the form of bio-enzymes is being explored by various researchers and field engineers^6–9^. These bio enzymes effectively stabilize soil while lowering the environmental impact of conventional stabilization techniques.

Bio-enzymes are protein molecules that catalyze chemical reactions in the soil to enhance the physical and engineering properties of treated soils. This takes place with the formation of a cementing bond that stabilizes the soil structure and reduces its affinity to water, and continue to function until there are no more reactions for them to catalyze^10,11^. They have also reported that micro-dose of enzymes is needed to stabilize large volumes of soil. Compared to the traditional stabilizers, bio-enzymes are economical, non-toxic, environmentally friendly, and organic in nature and are available in concentrated liquid form and are formulated using vegetable extracts^12^.

Enzymatic stabilizers are becoming popular, particularly in the field of highway construction^6,13–15^. Based on the observed performance of enzymatically stabilized subgrade soils for a few monsoon seasons, it has been reported by researchers and field engineers that enzymatic stabilization is successful for tropically weathered kaolinitic soils like red earth and lateritic soils used as subgrade soils in road construction projects^13,14,16–18^. The improvement in physical properties of enzymatically treated kaolinitic soils has been assessed through the Atterberg limits. A summary of the changes in Atterberg limits of enzyme treated kaolinitic soils as reported by various researchers has been presented in Table 1.

**Table 1:** Influence of terrazyme treatment on kaolinitic soils on Atterberg limits as reported in literature.

Sl No.	Liquid Limit, w_L_ (%)			Plasticity Index, I_*P*_ (%)			Source
	Natural soil	TerraZyme treated soil	Percent difference	Natural soil	TerraZyme treated soil	Percent difference	
1	35.0	27.6	21.1	10.0	3.6	64	^6^
2	27.0	19.5	27.8	3.8	3.5	7.9	^19^
3	39.0	35.0	10.3	17.0	17.0	0.0	^7^
4	42.1	39.8	5.5	23.5	22.2	5.5	^20^
5	42.2	38.2	9.5	19.4	12.2	37.1	^21^

The potential of using micro-dose of enzymes in improving the plasticity and engineering properties of enzyme treated kaolinitic soils (Red tropical soils) has been explored^3,8,9^ and hence, its application has been utilized in enhancing the stabilization effect of subgrade soils for highway application. However,  kaolinitic type of soils are not ubiquitously available in India and elsewhere. The alternate type of abundantly available soil is black cotton (B.C.) soil, which occurs mostly in the central and western parts of India and covers approximately 20% of the total area; and worldwide it is available in many countries like Australia, Sudan, Chad, Ethiopia, USA, and others^22^.

Though B.C. soil is available in abundance, its volumetric instability with variation in field moisture content due to the presence of expansive clay mineral, namely montmorillonite, makes its use in civil engineering applications undesirable. To date, no single method of stabilization has been found to be entirely effective in controlling the damages due to volumetric instability. With a need to utilize this type of expansive soils as an alternative to kaolinitic soils, researchers have tried to explore the possibility of using enzymatic stabilization on B.C. soils, which need effective stabilization to control the volumetric instability. A literature survey on the enzymatic stabilization of B.C. soils indicated that most of the researchers have reported the changes in plasticity properties of these soils. A summary of the reported changes in the plasticity properties of enzyme treated B.C. soils has been presented in Table 2. From the table, it can be observed that, in most of the studies, both the liquid limit and plasticity index of enzyme treated B.C. soils have reduced with ageing. In a few of the cases, the reduction (relative percentage reduction) in liquid limit is to the extent of 31.6%^23^ and 24.9%^24^, which seems to be quite significant. The authors of the study felt a need to ascertain the veracity of the effectiveness of enzyme treatment on the plasticity properties of B.C. soils as reported in the literature and extend the application of enzymatic stabilization for black soils to be used for various civil engineering applications.

**Table 2:** Influence of terrazyme treatment on B.C. Soils on Atterberg limits as reported in the literature.

Sl No.	Liquid Limit, w_L_ (%)			Plasticity Index, I_*P*_ (%)			Source
	Natural soil	TerraZyme treated soil	Percent difference	Natural soil	TerraZyme treated soil	Percent difference	
1	64.0	56.0	12.5	33.0	28.0	15.2	^25^
2	76.0	65.0	14.5	44.0	42.0	4.5	^7^
3	61.4	56.5	8.0	27.4	24.0	12.4	^26^
4	60.2	56.5	6.1	28.2	26.4	6.4	^27^
5	61.4	56.5	8.0	27.4	24.8	9.5	^28^
6	51.1	47.9	6.3	21.6	19.9	7.9	^20^
7	55.7	53.1	4.7	26.2	25.0	4.5	^20^
8	66.4	45.4	31.6	37.0	24.1	34.9	^23^
9	60.2	48.0	20.3	33.1	24.0	27.5	^29^
10	53.0	47.0	11.3	23.8	23.7	0.4	^30^
11	68.0	63.1	7.2	43.0	34.2	20.5	^31^
12	104.5	78.5	24.9	64.1	43.5	32.1	^24^

## Materials and methods

B.C. soils obtained from two different geological locations, namely Bijapur and Balekundri, Karnataka state, India, were used in this study. Henceforth, these two soils are referred to as S1 and S2, respectively. TerraZyme (TZ), which is available commercially and has also been successfully used in laboratory studies by various researchers^6,8,9^ as well as in field applications was used in the present study. However, two formulations of TZ namely, TZ 5X and TZ 11X were used in the present study. These are patented products from Nature (Plus) Inc., USA, and supplied by Avijeet Agencies Pvt. Ltd., India (sole distributors).

The selected soils were air dried before being pulverized with a wooden mallet to break up any clods, and later sieved through a 4.75 mm sieve. Using the standard methods outlined by the Bureau of Indian Standards (SP36)^32^, the physical and engineering properties of the selected soils, namely, specific gravity, liquid limit, plastic limit, shrinkage limit, particle size distribution, compaction characteristics, and undrained strength, were assessed, and the results are summarized in Table 3. Here it is to be noted that the undrained strength of soils can be determined either using an unconsolidated undrained triaxial shear test or in a simple way by unconfined compression strength test (UCS). In this study, the UCS test has been adopted for determining the undrained strength. Further, in this study, the Modified Proctor compaction test was adopted to evaluate the compaction characteristics. All these tests, except compaction characteristics were extended with TZ treatment as it was felt important to understand the effect of TZ on the physical and strength characteristics of B.C. Soils.

**Table 3:** Physical properties of the soils used in this investigation.

No.	Property	S1	S2	Relevant IS code^32^
1	Specific gravity	2.64	2.7	IS:2720 − 1980 (Part 3)^$^
2	Grain size distribution			IS:2720 − 1985 (Part 4)^$^
	Gravel (%)	2.5	0.9	
	Sand (%)	5.5	5.1	
	Silt (size) (%)	31	32	
	Clay (size) (%)	61	62	
3	Consistency limits			
	Liquid limit, w_L_ (%)	74.4	73.1	IS:2720 − 1985 (Part 5)^$^
	Plastic limit, w_P_ (%)	45.9	43.6	IS:2720 − 1985 (Part 5)
	Plasticity Index, I_p_ (%)	28.5	29.5	
	Shrinkage limit, w_S_ (%)	8.5	11.4	IS:2720 − 1985 (Part 6)^$^
	Shrinkage Index, I_s_ (%)	65.9	61.7	
4	Free swell test			IS:2720 − 1977(Part 40)^$^
	Sediment volume in water, V_d_ (cc)	15.5	21	
	Sediment volume in Kerosene, V_k_ (cc)	10	13	
	Free swell ratio (FSR)	1.6	1.6	
5	Compaction Characteristics			IS:2720 − 1983(Part 8)^$^
	Maximum dry density (Mg/m^3^)	1.5	1.5	
	Optimum moisture content (%)	24.5	25.8	
6	IS soil classification	MH	MH	

^$^All these codes are available as a compendium in Bureau of Indian Standards (SP36)^32^.

From Table 3, it is interesting to observe that both the physical and plasticity properties of the two B.C. soils selected for this study are quite similar though they are from different geological origins.

**Fig. 1:**
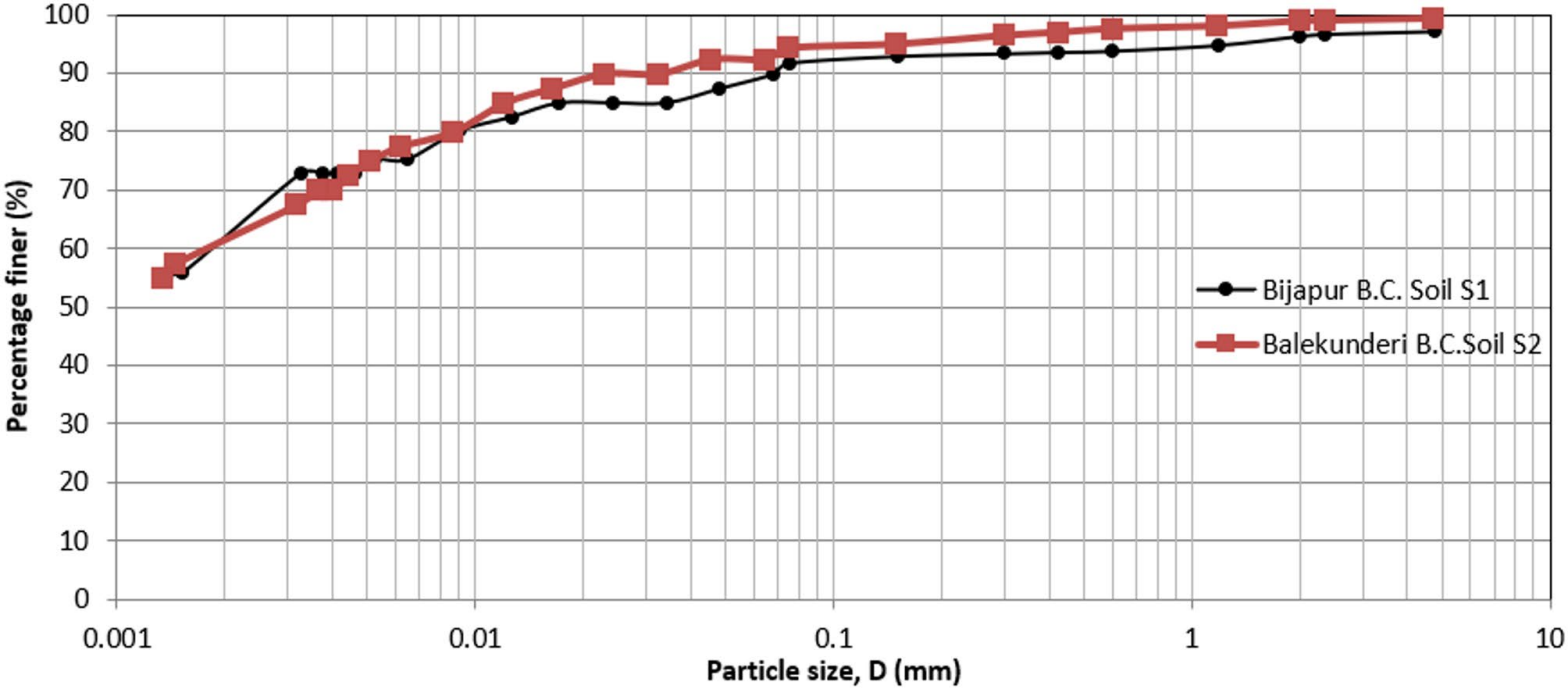
Plots of particle size distribution of B.C. soils used in the present study.

The plots of the grain size distribution of the B.C. soils used in this investigation are presented in Fig. 1.

**Fig. 2:**
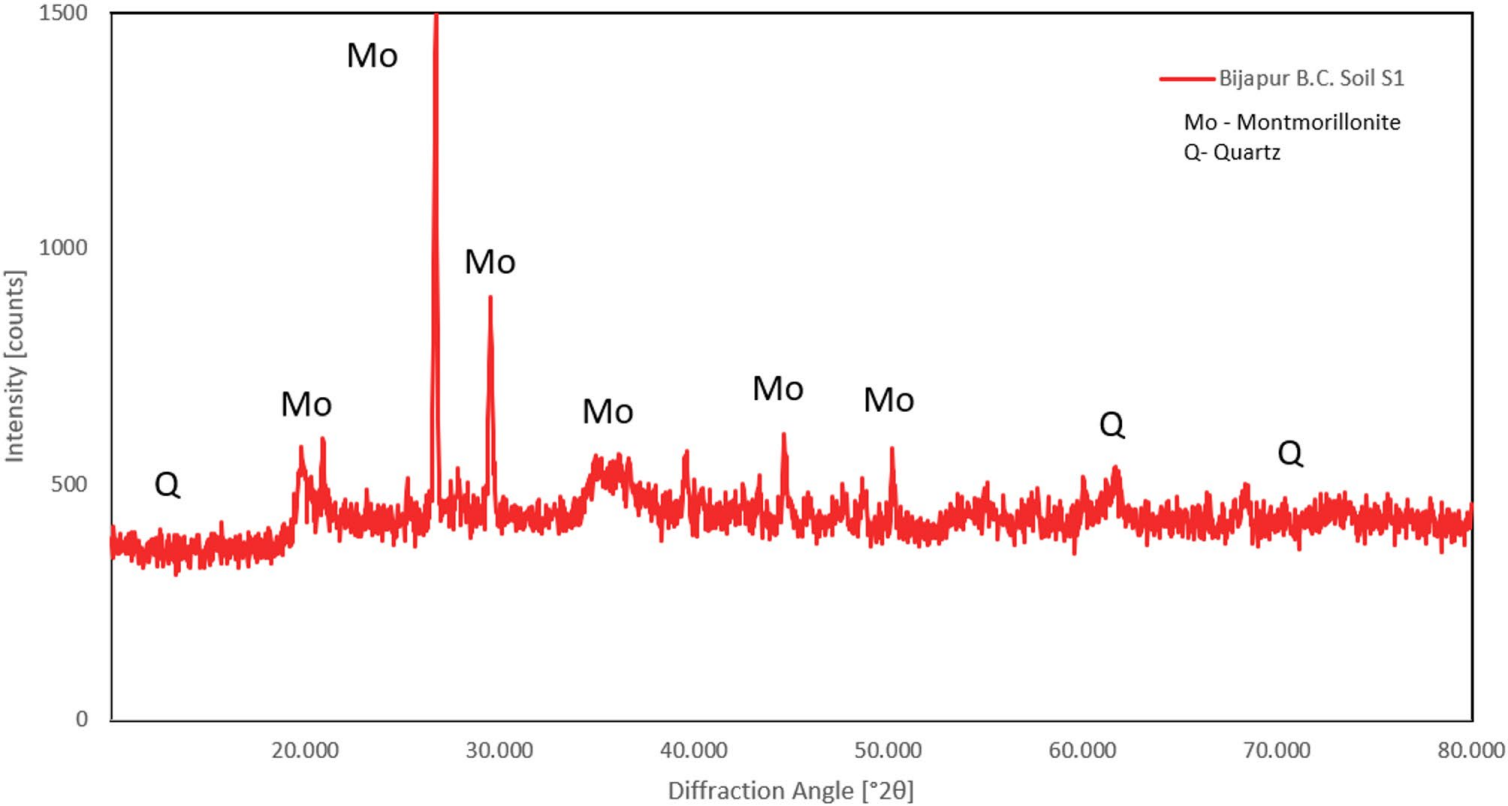
X-ray diffraction image of Bijapur B.C. soil S1.

**Fig. 3:**
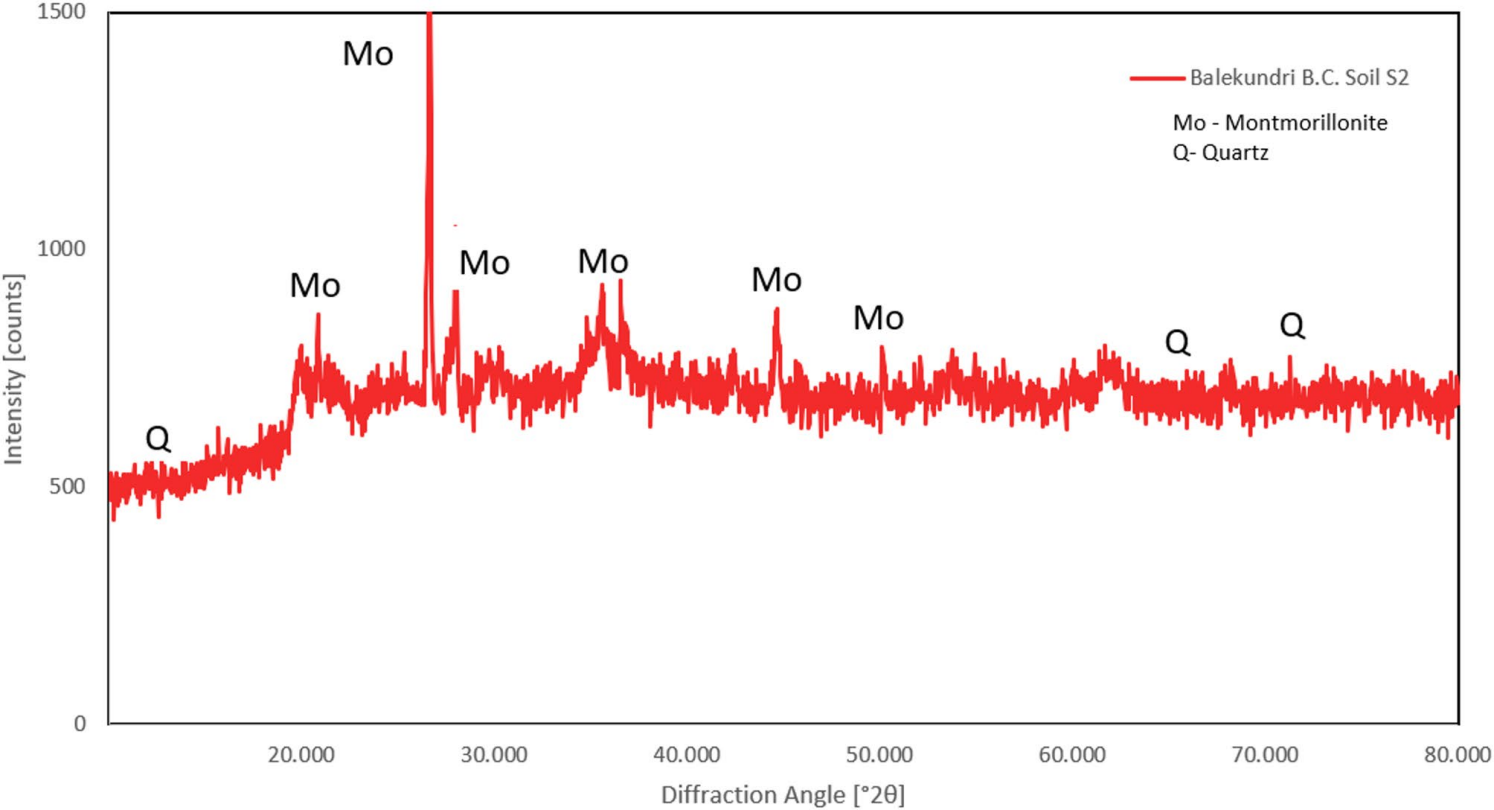
X-ray diffraction image of Balekundri B.C. soil S2.

The primary clay mineral present in the soils used in this study was identified using an X-ray diffractometer and Cu–kα radiation. Typical X-ray diffraction patterns of the soils are presented in Figs. 2 and 3.

The presence of the principal clay mineral for the two soils used in the present study was also ascertained indirectly through the free swell ratio (FSR)^33^. This has been used as a simple and alternate method of identifying the presence of the principal clay mineral present in fine-grained soils. FSR values of the soils used in this study are greater than 1.5, which indicates the presence of montmorillonite as the dominant clay mineral^33^.

Since, it was intended in this study to understand the influence of TerraZyme treatment on the plasticity properties and undrained strength of B.C. soils, scanning electron microscope (SEM) images of all the untreated and treated soils used in the present study were obtained using VEGA3 TESCAN Scanning Electron Microscope.

TerraZyme (TZ) is composed mostly of water (more than 50%), with the remaining portion being made up of fermented vegetable extract and ethoxylated alcohols, which are safer than any other enzymes in the category, namely, Perma-Zyme, Fujibeton, EarthZyme, Renolith, etc.,^34^. Variations in the amount of enzyme applied to the same soil result in different physical and engineering properties, suggesting that an inadequate amount of enzymes would not produce effective stabilization and that an excessive amount may not be beneficial^6^. Therefore, determining the most optimal TZ dosage would be beneficial for boosting the effectiveness of enzyme stabilization. As there are no established technical standards for the optimal dosage to be used, the empirical guidelines established by earlier researchers ^6,8,9,21^ is taken into consideration to determine the dosages for this study. The dosages listed in Table 4 were used for this investigation.

**Table 4:** Enzyme dosages utilized in the current investigation.

Dosage notation	ml/kg of soil
D1	0.065
D2	0.050
D3	0.039
D4	0.032

## Experimental program

An experimental program was formulated to determine the influence of TZ on the plasticity characteristics (liquid limit, plastic limit, and shrinkage limit) and unconfined compression strength of B.C. soils treated with enzymes. Past studies have shown that the stabilizing effect of TZ treatment can show observable changes in soil properties only after seven days, and that these changes can persist for up to two months^7,8,11,35,36^. So, to understand the influence of TZ on ageing, various ageing periods of 7, 15, 30, and 60 days were chosen for this study. For each dosage of TZ, four samples were prepared to study the influence of enzymatic stabilization for each of the ageing periods. Based on the four ageing periods planned for each dosage of TZ listed in Table 4, it was estimated that 1 kg of the selected soil passing through a 425 μm sieve would be sufficient for conducting experiments to evaluate the plasticity characteristics. The required TZ dosage was carefully extracted using a micropipette, and then injected into a beaker containing the estimated quantity of water, which when mixed with 1 kg of soil was sufficient to bring its consistency close to their respective liquid limit values. Each of the soil samples was mixed with the water added with selected dosage of TZ. Any additional water required for mixing was used to bring the consistency of the TZ treated soil slightly above the liquid limit. This mixture was thoroughly mixed with a spatula for over 30 min, and then transferred into polythene covers and labelled for future identification. To avoid moisture loss, these covers were properly sealed and kept in a desiccator for saturation before testing.

To determine the undrained strength characteristics of TZ treated B.C soils, cylindrical soil specimens of height 76 mm and diameter 38 mm were prepared at Modified Proctor maximum dry density and Optimum moisture content (OMC). TZ was added to the calculated amount of water needed to prepare soil specimens near to their OMC. Using weighed quantity of wet soil mix, identical cylindrical specimens were prepared using a static compactor. In order to prevent moisture loss and to enable the ongoing reaction between soil particles and TZ, the statically compacted specimens were carefully sealed in zip lock covers after being extruded from the mould and stored in a desiccator filled with water at the bottom to maintain humid condition within the desiccator. The samples were labelled for ease of identification and testing to determine the undrained strength with ageing at the selected ageing periods.

## Results and discussion

### B.C. Soil from Bijapur (S1 Soil)

The results of changes in the plasticity properties with ageing after treating it with TZ 5X formulation for the S1 soil are presented in Table 5.

**Table 5:** Plasticity properties of S1 soil with ageing with TZ 5X.

Ageing Period	Liquid Limit, w_L_ (%)	Plastic Limit w_*P*_ (%)	Plasticity Index, I_*P*_ (%)	Shrinkage Limit, w_S_ (%)	Shrinkage Index, I_S_ (%)
0 days	74.4	45.9	28.5	8.5	65.9
D1 TZ					
7 days	70.5	35.5	35.0	9.3	61.2
15 days	71.1	37.5	33.6	10.2	60.9
30 days	74.2	36.9	37.3	8.5	65.7
60 days	73.3	37.9	35.4	8.9	64.4
D2 TZ					
7 days	69.8	33.3	36.5	8.8	61.0
15 days	70.9	32.3	38.6	9.0	61.9
30 days	69.9	32.8	37.1	8.8	61.1
60 days	70.5	33.2	37.3	9.7	60.8
D3 TZ					
7 days	70.9	29.3	41.6	9.5	61.4
15 days	72.5	41.7	30.8	9.8	62.7
30 days	68.9	30.9	38.0	9.5	59.4
60 days	69.3	46.7	22.6	9.8	59.5
D4 TZ					
7 days	70.5	36.4	34.1	10.1	60.4
15 days	74.1	40.6	33.5	9.7	64.4
30 days	73.6	39.6	34.0	8.9	64.7
60 days	73.1	43.6	29.5	9.2	63.9

The natural soil S1 used in this investigation has a liquid limit of 74.4%. At the end of 60 days of ageing, the soil treated with TZ 5X at varying dosages of D1, D2, D3, and D4, the values of liquid limit were 73.3%, 70.5%, 69.3%, and 73.1%, respectively. It can be seen that TZ 5X has shown a decrease in liquid limit, which lies between 1.5% and 6.9%.

TZ treatment on plastic limit of S1 soil has a similar trend of decrease with ageing. The plastic limit of untreated S1 soil was 45.9%, whereas for D1, D2 and D4 dosages it was 37.9%,33.2%,43.6% respectively, showing a reduction in values of plastic limit of enzyme treated soil, lying between 5% and 27.7%; but for D3 dosage of TZ 5X, plastic limit was observed to be 46.7%, which is slightly more than the untreated soil, an increase of 1.7%.

**Fig. 4:**
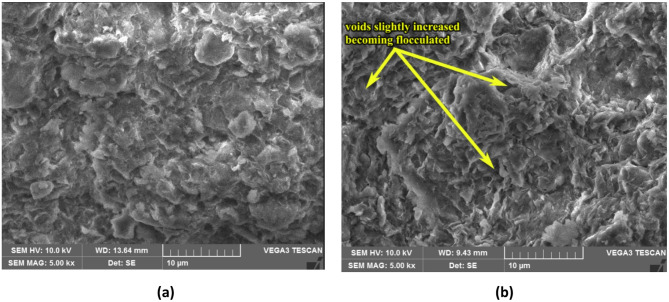
SEM view **(a)** Untreated S1 soil **(b)** S1 soil treated with D3 dosage of TZ 5X at the end of 60 days of ageing.

On the other hand, it is observed that shrinkage limit of S1 soil has increased with ageing by the addition of TZ 5X. The shrinkage limit at the end of the 60-days ageing period increased from 8.5% for the untreated soil to 8.9%, 9.7%, 9.8%, and 9.2% for dosages D1, D2, D3, and D4, respectively. The increase of shrinkage limit of enzyme treated soil lies between 4.7% and 15%, and D3 dosage of TZ 5X has a maximum of 15% increase in shrinkage limit (from 8.5% to 9.8%). It is commonly known that clays with a highly flocculated structure exhibit higher shrinkage limit in comparison to clays with a relatively dispersed structure. In general, higher the shrinkage limit of clay, the structure would tend to be more flocculated as compared to the clay having lower shrinkage limit. So, it can be inferred that an increase in shrinkage limit with TZ treatment, the clay structure would have probably changed from being a relatively more dispersed structure to a less dispersed structure indicating that the soil is tending to aggregate and form flocs. This is evident from the changes in clay structure as observed in the SEM views of the soil treated with D3 dosage TZ 5X at the end of 60 days of ageing as shown in Fig. 4. As discussed earlier, soil treated with dosage D3 has shown a significant increase (15% increase) in the values of shrinkage limit, and it is also evident from the slight change in soil structure as observed in the SEM view presented in Fig. 4b. Similar results of SEM views were observed for other dosages of TZ.

Shrinkage index (I_S_), which is the numerical difference between liquid limit and shrinkage limit has been used as index parameter to assess compressibility of a soil^37,38^. B.C. soils are not only expansive in nature, but also undergo significant compression on loading. Based on this consideration, the values of shrinkage index of S1 soil treated with TZ 5X with ageing were evaluated and the same is presented in Table 5. It can be observed that the shrinkage index has reduced from 65.9% from untreated condition to 64.4%, 60.8%, 59.5%, and 63.9% for dosages D1, D2, D3, and D4 respectively at 60 days of ageing. The reduction in values of I_S_ is in the range of 2.3% to 9.6%, the highest being for D3 dosage of TZ 5X. From these observations, it can be indirectly inferred that a clayey soil can become less compressible with ageing, if it is treated with an optimum dosage of TZ. From this it can be inferred that the soil treated with TZ 5X becomes comparatively more volumetrically stable.

### B.C. Soil from Balekundri (S2)

The results of changes in the plasticity properties with ageing after treating it with TZ 5X formulation for the S2 soil are presented in Table 6.

**Table 6:** Plasticity properties of S2 soil with ageing with TZ 5X.

Ageing Period	Liquid Limit, w_L_ (%)	Plastic Limit w_*P*_ (%)	Plasticity Index, I_*P*_ (%)	Shrinkage Limit, w_S_ (%)	Shrinkage Index, I_S_ (%)
0 days	73.1	43.6	29.5	11.4	61.7
D1 TZ					
7 days	80.6	37.7	42.9	8.8	71.8
15 days	90.9	36.2	54.7	9.2	81.7
30 days	73.4	36.3	37.1	8.7	64.7
60 days	89.1	52.2	36.9	8.0	81.1
D2 TZ					
7 days	79.3	42.0	37.3	8.8	70.5
15 days	87.7	48.9	38.8	7.8	79.9
30 days	76.4	37.1	39.3	8.4	68.0
60 days	87.9	45.4	42.5	7.9	80.0
D3 TZ					
7 days	78.4	42.5	35.9	8.9	69.5
15 days	93.5	56.4	37.1	9.1	84.4
30 days	93.3	47.7	45.6	8.1	85.2
60 days	73.7	44.3	29.4	7.9	65.8
D4 TZ					
7 days	78.2	44.2	34.0	9.0	69.2
15 days	92.7	54.8	37.9	9.2	83.5
30 days	81.9	46.4	35.5	9.4	72.5
60 days	74.0	44.4	29.6	8.5	65.5

Unlike S1 soil, adding TZ 5X to the S2 soil has shown to have an increase in the values of both liquid and plastic limits with ageing; whereas shrinkage limit has decreased with ageing.

The natural B.C. soil S2 used in this investigation has a liquid limit of 73.1%. On addition of TZ 5X to the soil, the liquid limit has increased with ageing instead of reduction as observed for S1 soil, and also being reported in the literature as summarized in Table 2. At the end of 60 days of ageing, for the soil treated with TZ 5X at varying dosages of D1, D2, D3 and D4 the values of liquid limit were observed to be 89.1%, 87.9%, 73.7% and 74.0%, respectively. It can be seen that liquid limit of treated soil with ageing has either increased substantially or remained very close to the initial value of untreated soil depending on the dosage of TZ, the percentage increase lying between 0.7% and 21.7%. This observation of increase in the values of liquid limit of enzyme treated soil with ageing is contradictory to that reported in the literature (Refer Table 2) and also as observed for S1 soil of the present study.

The influence of TZ 5X on plastic limit of treated S2 soil had a similar increasing trend with ageing as observed for liquid limit. The natural B.C. soil S2 has a plastic limit of 43.6%, whereas the plastic limit at varying dosages of D1, D2, D3 and D4 has values of 52.2%, 45.4%, 44.3% and 44.4%, respectively, showing an increase in plastic limit, the percentage increase lying between 1.3% and 16.3%. As the treated soil ages, it is evident that its plastic limit has either significantly increased or remained near to the original value of the untreated soil. This observation is also contradictory to that reported in the literature.

**Fig. 5:**
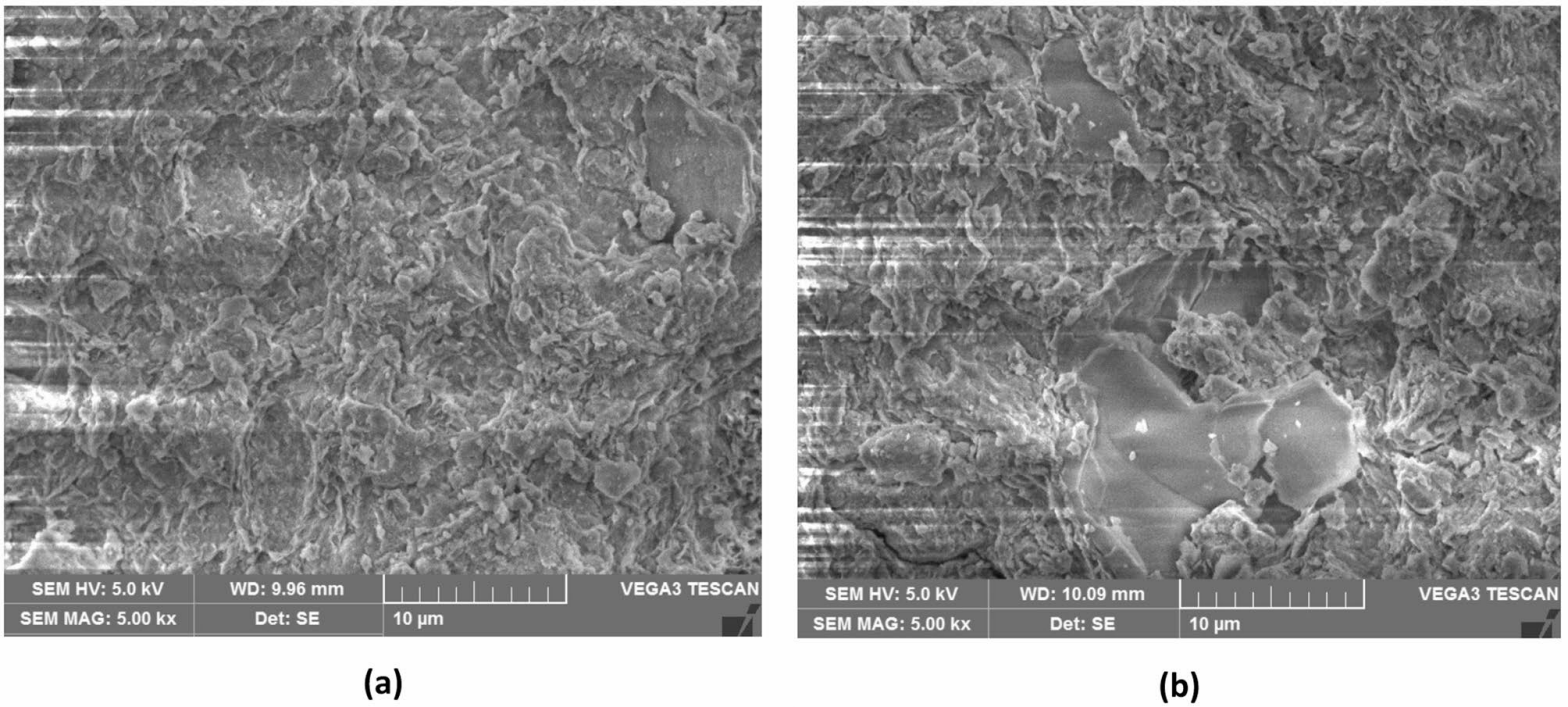
SEM view **(a)** Untreated S2 soil **(b)** S2 soil treated with D1 dosage of TZ 5X at the end of 60 days of ageing.

As compared to S1 soil, a decrease in shrinkage limit for all four dosages was observed for S2 soil with ageing. It is intriguing to observe that the liquid and plastic limits increased and shrinkage limit has decreased for each of the four TZ 5X dosages used in this study. A significant increase in both liquid limit and plastic limit, and decrease in shrinkage limit indicates that the soil is becoming relatively dispersed. However, these changes in the soil structure are not evidently observed from the SEM views of the untreated soil (Fig. 5a) and the treated soil (Fig. 5b) at the completion of the 60-day ageing period with TZ 5X. The reason for this is that montmorillonite clay mineral which is the predominant clay mineral present in B.C. soils has a strong diffuse double layer (DDL) because of its high surface activity, and hence has a dispersed structure. With TZ treatment it has become further relatively dispersed, and hence, the SEM views are quite similar. Similar results of SEM views were observed for other dosages of TZ.

The values of shrinkage index of S2 soil treated with TZ 5X with ageing were also evaluated and tabulated in Table 6. From the table, it can be observed that the shrinkage index has increased from 61.7% for untreated condition to 81.1%, 80.0%, 65.8%, and 65.5% for dosages D1, D2, D3, and D4 respectively at 60 days of ageing. The percentage increase in shrinkage index was in the range from 6% to 31.2%. Unlike S1 soil, values of shrinkage index for S2 soil have increased with ageing, indicating the soil S2 is likely to be more compressible with TZ treatment.

To verify the experimental findings reported in Table 6 for S2 soil, the entire exercise of evaluating the plasticity properties with ageing for various dosages of TZ was repeated. The results of this verification work have been summarized in Table 7.

**Table 7:** Plasticity properties of S2 soil with ageing with TZ 5X-Verification Trial.

Ageing Period	Liquid Limit, w_L_ (%)	Plastic Limit w_*P*_ (%)	Plasticity Index, I_*P*_ (%)	Shrinkage Limit, w_S_ (%)	Shrinkage Index, I_S_ (%)
0 days	73.1	43.6	29.5	11.4	61.7
D1 TZ					
7 days	75.7	41.2	34.5	8.2	67.5
15 days	78.8	40.0	38.8	10.1	68.7
30 days	74.8	40.8	34.0	9.7	65.1
60 days	86.6	46.5	40.1	9.8	76.8
D2 TZ					
7 days	74.1	40.4	33.7	8.0	66.1
15 days	77.0	45.9	31.1	9.4	67.6
30 days	73.6	40.0	33.6	9.3	64.3
60 days	85.4	44.2	41.2	9.6	75.8
D3 TZ					
7 days	70.4	40.7	29.7	10.8	59.6
15 days	79.8	51.3	28.5	8.9	70.9
30 days	78.8	44.2	34.6	9.2	69.6
60 days	74.5	46.9	27.6	9.3	65.2
D4 TZ					
7 days	70.2	40.2	30.0	10.9	59.3
15 days	80.8	49.5	31.3	11.0	69.8
30 days	76.8	44.9	31.9	10.1	66.7
60 days	75.7	45.7	30.0	10.0	65.7

Comparing the values of the plasticity properties between the two trials as observed from Tables 6 and 7, the values and trend of liquid limit, plastic limit, shrinkage limit and shrinkage index are more or less similar. This veracity of the above findings indicates that interaction of TZ with B.C soils is very unique and may be influenced by the various factors including the presence and amount of montmorillonite mineral, which needs to be verified from detailed studies.

From the results presented in the preceding paragraphs and the detailed discussions presented therein, it can be observed that contrasting changes in plasticity properties on enzymatic treatment with ageing is observed for two B.C. soils used in the present study with almost similar physical properties, but from different geological origin. With ageing on TZ treatment one soil (S1 soil) has shown a slight decrease in liquid limit, increase in shrinkage limit, and hence, a decrease in shrinkage index, indicating a probable volumetrically stable soil on treatment. Whereas, another soil (S2 soil) has shown quite opposite behavior, indicating a probable volumetrically unstable soil on treatment. This aspect needs to be further verified from detailed studies. It is possible that changes in the plasticity properties may not be the only indication of effectiveness of stabilization on enzyme treatment.

As the results with TZ 5X were not conclusive, a different formulation of the enzyme, namely TerraZyme 11X (TZ11X) which is preferred for soils having high clay content was procured to explore the possibility of obtaining more desirable changes in plasticity properties of B.C soils used in this study.

Second set of experiments were conducted using TZ11X following the same methodology explained in Sect. 3. The observed results of changes in plasticity properties with ageing after treatment with TZ11X for both S1 and S2 B.C. soils are tabulated in the Tables 8 and 9 respectively.

**Table 8:** Plasticity properties of Bijapur B.C. Soil (S1) with ageing with TZ 11X.

Ageing Period	Liquid Limit, w_L_ (%)	Plastic Limit w_*P*_ (%)	Plasticity Index, I_*P*_ (%)	Shrinkage Limit, w_S_ (%)	Shrinkage Index, I_S_ (%)
0 days	74.4	45.9	28.5	8.5	65.9
D1 TZ					
7 days	69.4	44.9	24.5	9.6	59.8
15 days	72.4	45.1	27.3	9.2	63.2
30 days	75.2	46.1	29.1	8.5	66.7
60 days	73.7	47.3	26.4	9.3	64.4
D2 TZ					
7 days	66.9	44.7	22.2	9.4	57.5
15 days	73.4	47.7	25.7	9.7	63.7
30 days	71.6	45.4	26.2	7.8	63.8
60 days	71.6	45.1	26.5	8.9	62.7
D3 TZ					
7 days	69.7	46.2	23.5	10.1	59.6
15 days	73.3	42.7	30.6	9.8	63.5
30 days	70.9	45.5	25.4	8.3	62.6
60 days	69.4	46.8	22.6	9.2	60.2
D4 TZ					
7 days	69.4	39.7	29.7	10.3	59.1
15 days	70.9	43.6	27.3	9.4	61.5
30 days	73.9	45.2	28.7	8.6	65.3
60 days	74.2	47.1	27.1	9.2	65.0

At the end of 60 days of ageing, the S1 soil treated with TZ 11X at varying dosages of D1, D2, D3, and D4, the values of liquid limit were 73.7%, 71.6%, 69.4%, and 74.2%, respectively. The percentage reduction of liquid limit of enzyme treated soil lies between 0.3% and 6.9%. As observed from Table 8, for S1 soil for all dosages of TZ11X, initially there was a decrease in liquid limit by the end of 7 days ageing. With further ageing, an increase in liquid limit was observed, which however, did not exceed the value of the liquid limit of untreated soil.

TZ11X treatment on plastic limit of S1 soil has shown to increase with ageing. The plastic limit of untreated S1 soil was 45.9%, whereas for D1, D2, D3 and D4 dosages was 47.3%,45.1%,46.8% and 47.1% respectively. This behavior is contrary to the changes observed with TZ 5X. The reason for this behavior has to be explored with further studies.

**Fig. 6:**
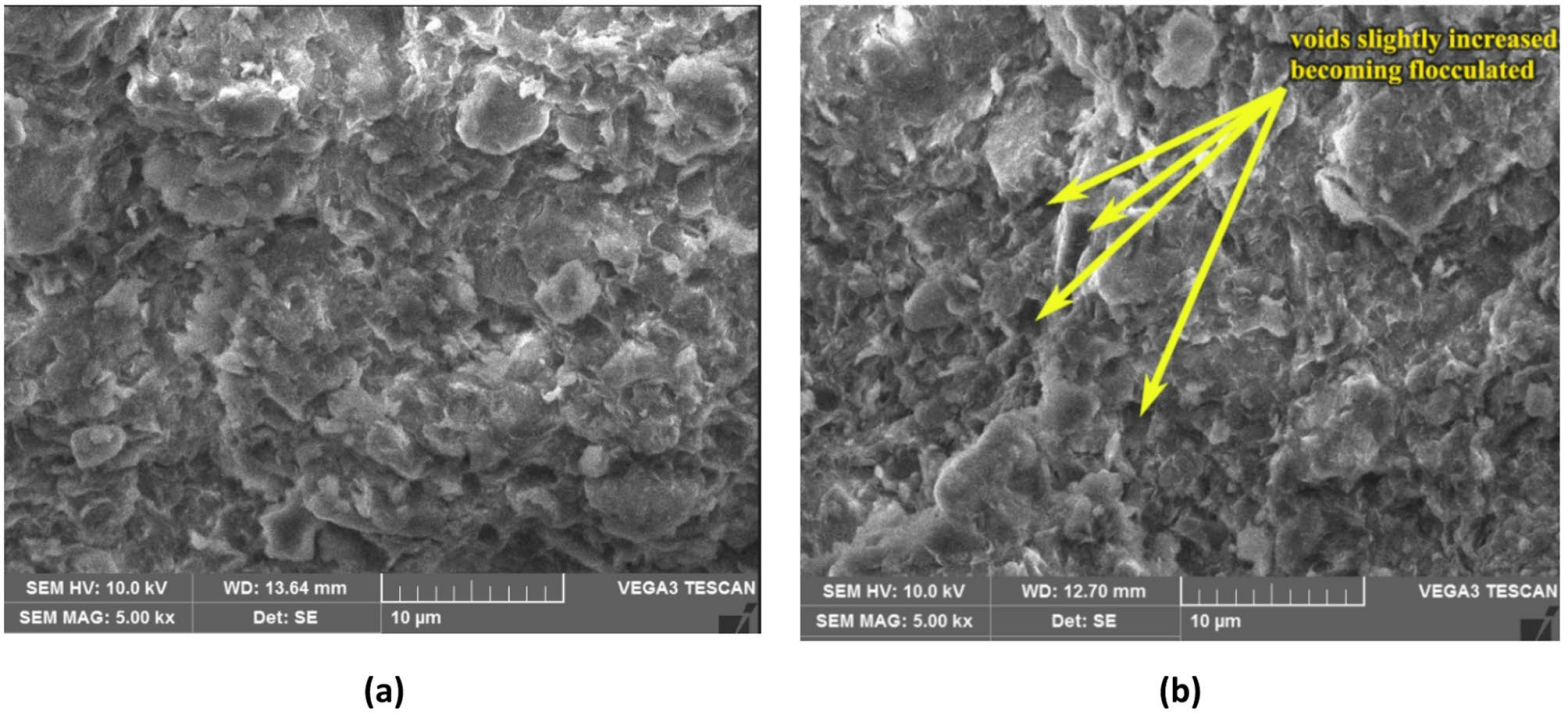
SEM view **(a)** Untreated S1 soil **(b)** S1 soil treated with D3 dosage of TZ 11X at the end of 60 days of ageing.

It is observed that shrinkage limit of S1 soil has increased with ageing by the addition of TZ11X. The shrinkage limit at the end of the 60-days ageing period increased from 8.5% for the untreated soil to 9.3%, 8.9%, 9.2% and 9.2% for dosages D1, D2, D3, and D4, respectively. The percentage increase in shrinkage limit after enzyme treatment lies between 4.7% and 9.4%. Thus, the increase in shrinkage limit of soils treated with TZ11X with ageing suggests that the soil is getting more flocculated. A notable alteration in soil structure can be seen in the SEM view displayed in Fig. 6b, (typical view for one dosage only), which is for soil treated with dosage D3 when compared to Fig. 6a, which is untreated soil. This shows a reflection on the changes in the plasticity characteristics including the shrinkage index values, being the least for this dosage of TZ. Similar results of SEM views were observed for other dosages of TZ.

Table 9 shows the changes in plasticity characteristics with ageing after treating the S2 soil with the TZ 11X formulation.

**Table 9:** Plasticity properties of Balekundri B.C. Soil (S2) with ageing with TZ 11X.

Ageing Period	Liquid Limit, w_L_ (%)	Plastic Limit w_*P*_ (%)	Plasticity Index, I_*P*_ (%)	Shrinkage Limit, w_S_ (%)	Shrinkage Index, I_S_ (%)
0 days	73.1	43.6	29.5	11.4	61.7
D1 TZ					
7 days	75.9	50.5	25.4	6.5	69.4
15 days	74.4	46.2	28.2	13.8	60.6
30 days	77	49	28	14.9	62.1
60 days	76.5	49.6	26.9	16.3	60.2
D2 TZ					
7 days	73.1	45.9	27.2	6.6	66.5
15 days	72.4	47.4	25	13.6	58.8
30 days	74.5	50.1	24.4	13.8	60.7
60 days	73.6	44.9	28.7	14.2	59.4
D3 TZ					
7 days	61.9	32.4	29.5	14.1	47.8
15 days	73.3	46.7	26.6	14.1	59.2
30 days	74.1	47.9	26.2	15.9	58.2
60 days	77.4	49.6	27.8	15.6	61.8
D4 TZ					
7 days	61.8	30.8	31	13.6	48.2
15 days	75.5	47.3	28.2	14.2	61.3
30 days	75	48.7	26.3	14.9	60.1
60 days	77.1	49.3	27.8	15.4	61.7

S2 soil when treated with TZ 11X has shown to have an increase in the values of liquid limit for all dosage of TZ, which is similar to the behaviour observed with TZ 5X. At the end of 60 days of ageing, the S2 soil treated with TZ 11X at varying dosages of D1, D2, D3, and D4, the values of liquid limit were 76.5%, 73.6%, 77.4%, and 77.1%, respectively. The increase in the values of liquid limit of enzyme treated soil lies between 0.7% and 5.8%.

Similarly, the plastic limit of TZ 11X treated soil had also increased with ageing. The natural B.C. soil S2 has a plastic limit of 43.6% and the plastic limit at varying dosages of D1, D2, D3 and D4 has values of 49.6%, 44.9%, 49.6% and 49.3%, respectively. The percentage increase of plastic limit of enzyme treated soil lies between 2.7% and 13.5%.

**Fig. 7:**
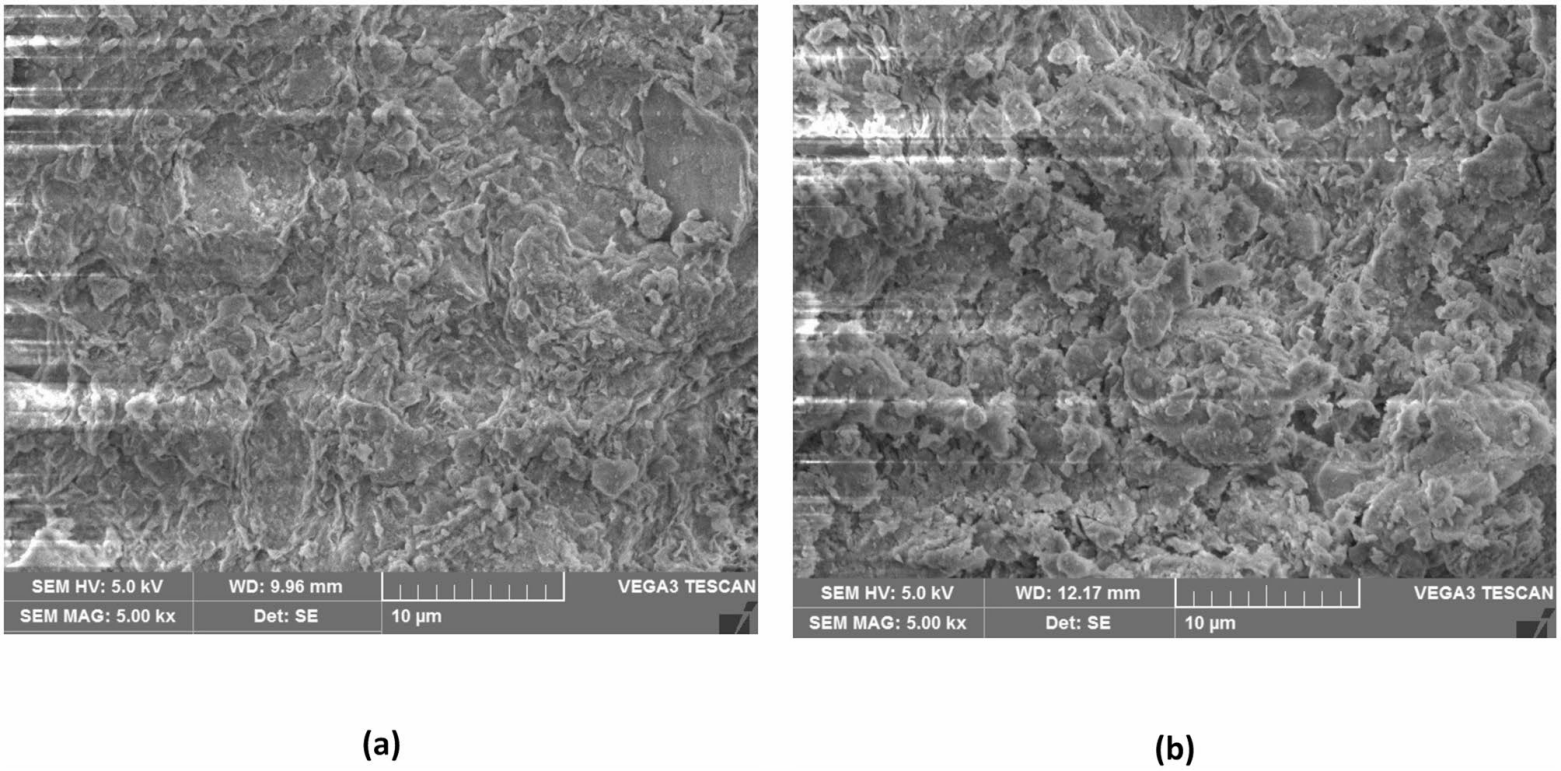
SEM view **(a)** Untreated S2 soil **(b)** S2 soil treated with D1 dosage of TZ 11X at the end of 60 days of ageing.

For S2 soil, unlike with TZ 5X, an increase in shrinkage limit was observed with TZ 11X treatment. The shrinkage limit of untreated soil was 11.4%. At the end of the 60-day ageing period, the shrinkage limit for the dosages D1, D2, D3, and D4 was 16.3%, 14.2%, 15.6%, and 15.4% respectively. The percentage increase in shrinkage limit was between 24.5% and 43%. It can be inferred that the soil treated with TZ11X is becoming less dispersed when compared to the soil treated with TZ 5X, which can be observed from Fig. 7a and b (typical views for one dosage only). The shrinkage limit of treated soil with dosage D1 has increased significantly. Similar results of SEM views were observed for other dosages of TZ.

It is evident that the two B.C. soils, S1 and S2, did not react similarly to treatments with TZ 5X and 11X at the same dosages and ageing periods.

As index properties are inferential properties of soils, changes in plasticity properties by using TZ may not be a true reflection on their strength properties. Therefore, evaluation of undrained strength properties of soil on TZ treatment was necessitated due to fact that TZ has been successfully used in stabilizing B.C. soils of highway subgrades as reported with case studies in the website of the supplier of TZ. However, the case studies do not document the details of the changes in either their plasticity properties or strength properties. Similar improvement in undrained strength of B.C soils with other bio enzymes has been reported in the literature^39,40^. So, the authors of the study felt the need to explore the influence of TZ treatment on the undrained strength properties of B.C soils with ageing to bring out the appropriate mechanism/s through which stabilization is being affected as reflected in the improvement in undrained strength. The results of such an attempt have been reported in the ensuing section.

### Unconfined compressive strength (UCS)

The experimental tests results of UCS test carried on B.C. soils to study the influence of TZ are summarized in Tables 10 and 11 for S1 soil, respectively treated with TZ 5X and 11X formulations; and Tables 12 and 13 for S2 soil, respectively treated with TZ 5X and 11X formulations. Here the ratio of improvement in UCS on enzyme treatment is expressed as improvement factor (IF)^40^.

**Table 10:** UCS of Bijapur B.C. Soil (S1) with ageing with TZ 5X.

Ageing Period	D1 TZ	IF	D2 TZ	IF	D3 TZ	IF	D4 TZ	IF
7 days	303.5^*^	1.04	306.2	1.05	327.3	1.12	319.9	1.09
15 days	307.9	1.05	319.3	1.09	347.9	1.19	323.9	1.11
30 days	309.4	1.06	327.5	1.12	368.9	1.26	343.7	1.17
60 days	311.2	1.06	333.9	1.14	395.4	1.35	358.3	1.22

*UCS values are in kPa.

**Table 11:** UCS of Bijapur B.C. Soil (S1) with ageing with TZ 11X.

Ageing Period	D1 TZ	IF	D2 TZ	IF	D3 TZ	IF	D4 TZ	IF
7 days	295.8^*^	1.01	312.4	1.07	345.5	1.18	321.6	1.10
15 days	309.7	1.06	325.9	1.11	370.7	1.27	340.1	1.16
30 days	311.9	1.06	342.6	1.17	396.5	1.35	377.6	1.29
60 days	333.7	1.14	349.9	1.19	437.0	1.49	384.8	1.31

*UCS values are in kPa

**Table 12:** UCS of Balekundri B.C. Soil (S2) with ageing with TZ 5X.

Ageing Period	D1 TZ	IF	D2 TZ	IF	D3 TZ	IF	D4 TZ	IF
7 days	260.9^*^	1.03	244.6	0.97	257.2	1.02	254.8	1.01
15 days	269.8	1.07	268.5	1.07	272.3	1.08	267.2	1.06
30 days	284.9	1.13	289.9	1.15	296.2	1.17	300.0	1.19
60 days	327.8	1.30	310.1	1.23	315.2	1.25	317.7	1.26

*UCS values are in kPa.

**Table 13:** UCS of Balekundri B.C. On soil (S2) with ageing with TZ 11X.

Ageing Period	D1 TZ	IF	D2 TZ	IF	D3 TZ	IF	D4 TZ	IF
7 days	267.1^*^	1.06	233.9	0.93	231.9	0.92	252.5	1.00
15 days	266.5	1.06	281.0	1.11	298.1	1.18	268.5	1.07
30 days	292.5	1.16	297.9	1.18	314.2	1.25	316.6	1.26
60 days	367.2	1.46	318.9	1.26	332.9	1.32	333.6	1.32

*UCS values are in kPa

With TZ treatment of the soils with both formulations (5X and 11X), the values of UCS have improved significantly. Though the usage of TZ showed a diverse behaviour with respect to changes in the liquid limit for the two B.C. soils (S1 and S2 soils) of similar physical properties used in the present study as compared to a decrease in liquid limit reported in the literature and summarized in Table 2, an increase in UCS with ageing was observed with TZ for both S1 and S2 soils. For S1 soil, at 60 days of ageing with TZ 5X, it is observed that D3 dosage has given the highest change in undrained strength, being 35% increase (from 292.9 kPa to 395.4 kPa) and for the same dosage, an increase of 49.2% (from 292.9 kPa to 437.0 kPa) in UCS was observed with TZ 11X. Similarly, for S2 soil sample with a TZ dosage of D1, a 30% increase in UCS was observed with TZ 5X (from 252.1 kPa to 327.8 kPa) and for the same dosage, a 45.6% increase in UCS was observed with TZ 11X (from 252.1 kPa to 367.2 kPa).

Similar increase in the values of UCS on enzyme treatment has been observed and reported in the literature by various researchers as summarized in Tables 14 and 15 respectively for kaolinitic soils and montmorillonitic soils (B.C. soils). Further, it can be observed that improvement factor (IF) is found to vary from 2.2 to 22.4 for kaolinitic soils and 1.2 to 10.4 for B.C soils. In the present study, S1 soil treated with D3 dosage of TZ, has highest IF of 1.35 and 1.49 for TZ 5X and TZ 11X respectively and for S2 soil, with a TZ dosage of D1, has highest IF of 1.30 and 1.46 for TZ 5X and TZ 11X respectively. This shows that improvement in UCS is quite possible on TZ treatment to both soil types and it can be quite variable.

**Table 14:** Influence of enzyme on UCS of kaolinitic soils (Red earths) as reported in literature.

Unconfined compression strength (kPa)			Source
Untreated	Enzyme treated	Improvement Factor (IF)	
142.0	782.0	5.51	^6^
414.0	3841.0	9.28	^7^
2.3	5.1	2.22	^20^
25.0	560.0	22.40	^21^
250.4	580.9	2.32	^40^

**Table 15:** Influence of enzyme on UCS of montmorillonitic soils (B.C. soils) as reported in literature.

Unconfined compression strength (kPa)			Source
Untreated	Enzyme treated	Improvement Factor (IF)	
267.0	859.0	3.22	^25^
147.0	1533	10.43	^7^
3.53	8.86	2.51	^26^
3.57	8.72	2.44	^27^
3.53	8.86	2.51	^28^
2.28	5.67	2.49	^20^
2.68	6.53	2.44	^20^
52.9	125.7	2.38	^23^
127.48	154.04	1.21	^29^
127.52	210.32	1.65	^41^
210.0	320.0	1.52	^39^
-	-	1.28^#^	^42^ #Information of increase in UCS of 28% is available from the source, based on which IF is approximated
151.14	787.0	5.20	^40^
90.0	135.0	1.50	^24^

### Proposed mechanism for improvement in undrained strength

Though various researchers have reported an improvement in UCS after enzyme treatment as presented in Tables 14 and 15, very few have tried to bring out the possible mechanism/s to explain the observed behavior in the laboratory studies, notable among them are Scholen^10^, Marasteanu et al.^12^, Tingle et al.^11^, and Pooni et al.^42^.

Scholen^10^ and Marasteanu et al.^12^, proposed that enzymatic treatment leads to a reduction in the thickness of the diffused double layer, which involves the clay lattice adsorbing the enzymes in the treated soil and releasing cations in a method similar to cation exchange. The affinity of clay to moisture thereby reduces as large organic molecules in the soil may bond with the enzymes, which would then be drawn to the net negative surface charge of the clay particles neutralizing their negative charge. This leads to reduced interconnectivity of voids. A similar reduction in affinity for water was reported by Tingle et al.^11^, as a possible mechanism to explain the changes observed on TerraZyme treatment in soils.

Pooni et al.^42^, have proposed three possible changes due to enzyme interaction with the clay particles. Firstly, there will be a change in affinity for water. Secondly, reduction in ingress of water within the soil. Thirdly, an improvement in density of the enzyme treated soil sample with ageing leading to an increase in strength. However, considering the way identical samples are prepared in laboratory studies, maintaining appropriate initial conditions of preparation with enzyme treatment to evaluate the strength changes with ageing, there will be no possibility of change in the mass of the sample after its preparation. Hence, there will be no possibility in the improvement in density of the laboratory prepared sample with ageing to influence the enzyme treated soil samples to show a significant increase in UCS as reflected in the values of IF of both in the present study as well as that is reported in the literature. Similar is the case of improvement in UCS of enzyme treated soils in the field applications. Based on this consideration, the proposed mechanism of improvement in density by Pooni et al.^42^, is not appropriate to explain the increase in strength of enzyme treated soil specimens with ageing.

As discussed earlier, based on the SEM views presented through Figs. 4, 5, 6 and 7, it can be observed that enzyme treatment has a notable influence on the soil structure at an optimum dosage of enzyme treatment. Continuing with this consideration, the authors of the present study propose the following mechanisms to explain the increase in UCS of enzyme treated B.C. soils.

- Reduction in thickness of diffuse double layer of the clay particles leading to an alteration in soil structure from a highly dispersed to relatively less dispersed structure, causing the soil to flocculate as inferred by an increase in shrinkage limit.
- Increase in undrained strength due to a relatively stable flocculated structure aided with induration/bonding at the contact points of clay particles.

Further, to substantiate the above discussion, untreated B.C soil which has a dispersed structure as presented in Fig. 4a (S1 soil) and Fig. 5a (S2 soil), after enzyme treatment, the clay structure is observed to change to a relatively less dispersed structure as presented through SEM views through Figs. 4, 5, 6 and 7. The optimum dosage of enzyme treatment has resulted in more conspicuous change in soil structure as reflected in the SEM views and also presented in the earlier discussion.

From the above discussion, the proposed mechanisms appropriately explain the role of enzyme treatment in improving the undrained strength of B.C. soils.

## Conclusions

This study is an attempt done to understand the influence of TZ on the plasticity and undrained strength properties of B.C soils. Important observations emerging from the study are summarized below.

1. As observed in the literature, most of the studies of enzyme treatment on B.C. soils have reported a significant decrease in liquid limit with ageing. However, the results of the present study show that liquid limit has slightly decreased (maximum of 6.9%) for S1 soil with ageing after treatment with TZ 5X, whereas S2 soil has shown a substantial increase (maximum of 22%) in liquid limit with ageing with TZ5X. Further, with TZ11X, the observed changes in liquid limit is similar to that observed with TZ5X. However, the increase in liquid limit for S2 soil is less (maximum of 6%) as compared to that observed with TZ5X.
2. However, the influence of TZ treatment on shrinkage limit with ageing has shown diverse results for the two B.C soils used in the study.
3. The observations of plasticity properties at the macro level were confirmed by the changes at the microlevel as observed through the SEM views.
4. However, the results of undrained strength with enzyme treatment using both the formulations of TZ (5X and 11X) have shown to improve with ageing as compared to the untreated B.C soils used in the study. Further, it was observed that TZ 11X has shown a marked improvement in strength as compared to TZ 5X. At optimum dosage of the enzymes, it was observed that with TZ 5X, there was 35% increase in the undrained strength for S1 soil and 30% increase for S2 soil. Further, with TZ 11X, the corresponding increase in the undrained strength was 49.2% and 45.6% respectively for S1 and S2 soils.
5. Two mechanisms are proposed to explain the observed changes in plasticity properties and undrained strength of TerraZyme treated B.C soils with ageing: (i) Change in clay structure due a reduction in thickness of diffuse double layer of the clay particles causing the particles to flocculate as inferred by an increase in shrinkage limit. (ii) Increase in undrained strength due to a relatively stable flocculated structure aided with induration/bonding at the contact points of clay particles.

From the results of the present study, it can be inferred that the influence of TZ on the plasticity properties of enzyme treated B.C soils may not be a true reflection of the effectiveness of enzyme on strength properties, and hence, stabilizing effect on B.C. soils. This further suggests that in situations where B.C. soils are to be treated with enzymes, it is advisable to carry out detailed testing to evaluate the suitable formulation and dosage of TerraZyme and use the same for field application.

## Data Availability

The datasets used and/or analyzed during the current study available from the corresponding author on reasonable request.

## Abbreviations

B.C.: Black cotton
DDL: Diffuse double layer
FSR: Free swell ratio
IF: Improvement factor
I_P_: Plasticity index
I_S_: Shrinkage index
OMC: Optimum moisture content
S1 soil: Bijapur black cotton soil
S2 soil: Balekundri black cotton soil
SEM: Scanning electron microscope
TZ: TerraZyme
UCS: Unconfined compressive strength
w_L_: Liquid limit
w_P_: Plastic limit
w_S_: Shrinkage limit
